# Active-distributed temperature sensing dataset beneath a braided river

**DOI:** 10.1016/j.dib.2023.109756

**Published:** 2023-11-04

**Authors:** Alice J. Sai Louie, Leanne K. Morgan, Eddie W. Banks, David Dempsey, Scott Wilson

**Affiliations:** a Waterways Centre for Freshwater Management, University of Canterbury, Private Bag 4800, Christchurch 8140, New Zealand; b National Centre for Groundwater Research and Training and College of Science and Engineering, Flinders University, GPO Box 2100, Adelaide, SA 5001, Australia; c Department of Civil and Natural Resources Engineering, University of Canterbury, Private Bag 4800, Christchurch 8140, New Zealand; d Lincoln Agritech Limited, PO Box 69 133, Lincoln, Christchurch 7640, New Zealand

**Keywords:** Fiber optics, Heat as a tracer, River, Groundwater recharge, Alluvial aquifer

## Abstract

Braided rivers play a significant role in replenishing groundwater, but our understanding of how these recharge rates fluctuate over time remains limited. Traditional techniques for gauging groundwater recharge are ineffective for studying complex braided river systems due to their insufficient spatiotemporal resolution. To address this gap, active-distributed temperature sensing (A-DTS) was used. This method combines fiber optic temperature measurements with an active heat source, enabling quantification of groundwater fluxes. In this study, twelve consecutive A-DTS surveys were conducted on a 100 m long hybrid fiber optic cable to a depth of 5 m beneath the Waikirikiri Selwyn River. This experiment was conducted during a period of relatively stable river stage and flow, highlighting the effectiveness of using A-DTS to measure temporal changes in groundwater recharge.

Specifications Table

**Table:** 

Subject	Water Science and Technology
Specific subject area	The Waikirikiri Selwyn River is a braided rivers system located in Canterbury in the South Island of New Zealand. Its headwaters start in the Southern Alps and travels 93 km across the Canterbury Plains towards the Pacific Ocean (Fig. 1). The study site is located within an ephemeral reach of this braided river system. The river is an important recharge mechanism to the highly transmissive Canterbury Plains aquifer systems. The study site comprises two active channels separated by a gravel bar at an elevation of approximately 210 m above sea level.
Data format	.xlsx file containing time series of temperature data versus distance along the cable.
Type of data	Table
Data collection	Horizontal directional drilling was used to construct a 100 m long 125-mm diameter drillhole to a depth of approximately 5 m beneath the Waikirikiri Selwyn River. A temporary 125-mm diameter poly pipe casing was installed to provide a conduit for a hybrid fiber optic cable to be positioned within the drillhole. The temporary casing was removed and the drillhole collapsed around the cable. This was used to conduct a 24-h active-distributed temperature sensing (A-DTS) experiment using a Silixa XT-DTS combined with a Silixa Heat Pulse System. Twelve heat pulse cycles were conducted and involved applying a 1-h heat pulse followed by 1-h of measuring the temperature recession before the next cycle.
Data source location	• Region: Canterbury • Country: New Zealand • Latitude and longitude are shown on the study site map
Data accessibility	Repository name: Mendeley data Data identification number: DOI: 10.17632/w2kr9m6s5h.2 Direct URL to data: https://data.mendeley.com/datasets/w2kr9m6s5h/2

## 1 Value of the Data

• Globally, river loss is a significant contributor to groundwater recharge [2]; however, this process is not well understood in braided rivers [3]. Braided river systems are dynamic, heterogeneous environments, with complex interactions between surface water and groundwater, making them challenging subjects for scientific study [3]. Recently, we have quantified how groundwater recharge from braided rivers varies spatially [1]. This data is valuable because it provides an insight into how groundwater recharge from a braided river varies temporally.
• This comprehensive 24-h A-DTS dataset is significant because it provides a methodology to be able to calculate groundwater recharge beneath a braided river. The dataset includes temperature measurements in the saturated zone beneath the river, between 31.56 m and 103.59 m along the cable (i.e., beneath the water table of the shallow aquifer under the river), and the unsaturated zone either side of the river. The data is sampled at 2-h increments comprising twelve consecutive A-DTS surveys over a 24-h experiment.
• This dataset was collected using innovative field techniques [1]. The implementation of horizontal directional drilling to install a cable beneath the active river channels of the Waikirkiri Selwyn River is novel [1]. This contains a hybrid fiber optic (FO) cable, containing both FO cables used for temperature sensing as well as copper cables for heating [4]. The use of the Silixa XT-DTS distributed temperature sensor, with a resolution of 0.01 °C, combined with a Silixa Heat Pulse System, used to heat the cable with 15 W m^−1^, offers an advanced measurement technique to calculate groundwater velocities [4].
• The configuration of the fiber optic cable used a double-ended differential loss correction method, resulting in temperature measurements recorded at 12 s increments. Each survey consisted of 300 s measuring the ambient temperature, a 3600 s heating period, followed by 3300 s measuring the temperature recession. This sampling routine effectively captured temperature changes along the cable and also provided a sufficient increase in temperature from the ambient temperature conditions before returning to the baseline temperature within each survey.
• Students and researchers can use this dataset to learn how to process A-DTS data and use an analytical solution to calculate groundwater fluxes and thermal properties of the materials surrounding the cable.
• Gaining insights into how braided river systems recharge the underlying aquifer is important for river and groundwater management. This knowledge can inform decisions related to river management practices, water resource planning, conservation, and sustainable water use.

## 2 Data Description

This dataset contains raw A-DTS temperature measurements from the 24-hsurvey conducted from 14:00 2 September to 14:00 3 September 2022. The structure of the dataset contains a row for each individual observation point along the cable, and columns for each timestamp within the dataset. The top row of the spreadsheet, row 1, contains the datetime stamp for each temperature measurement (format d/mm/yyyy h:mm:ss). Temperature measurements are recorded at a temporal resolution of every 12 s, such that each column in the spreadsheet contains temperature measurements taken along the entire length of the cable at 12 s intervals. The first row of the spreadsheet, row A, entitled ‘Distance’ contains the distance along the cable, including the 33 m section of cable above ground in the calibration baths. the second row of the spreadsheet, Row B, entitled ‘distance_corrected’ contains the corrected distance along the cable consisting of only the length of the cable that is buried beneath the ground. The temperature measurements are at 0.254 m intervals; hence each subsequent row of data contains temperature measurements taken 0.254 m apart. The spatial resolution of the temperature measurement is 0.54 m and a temperature resolution of 0.01 °C. The spatial resolution is greater than the sample interval and is estimated by applying the 90% step change method [5,6].

A shape file is provided showing the cable location and ground surface coordinates beneath the river. The distance along the cable, starting at the southwest end of the cable, corresponds to the ‘distance corrected’ values provided in the spreadsheet.

**Fig. 1:**
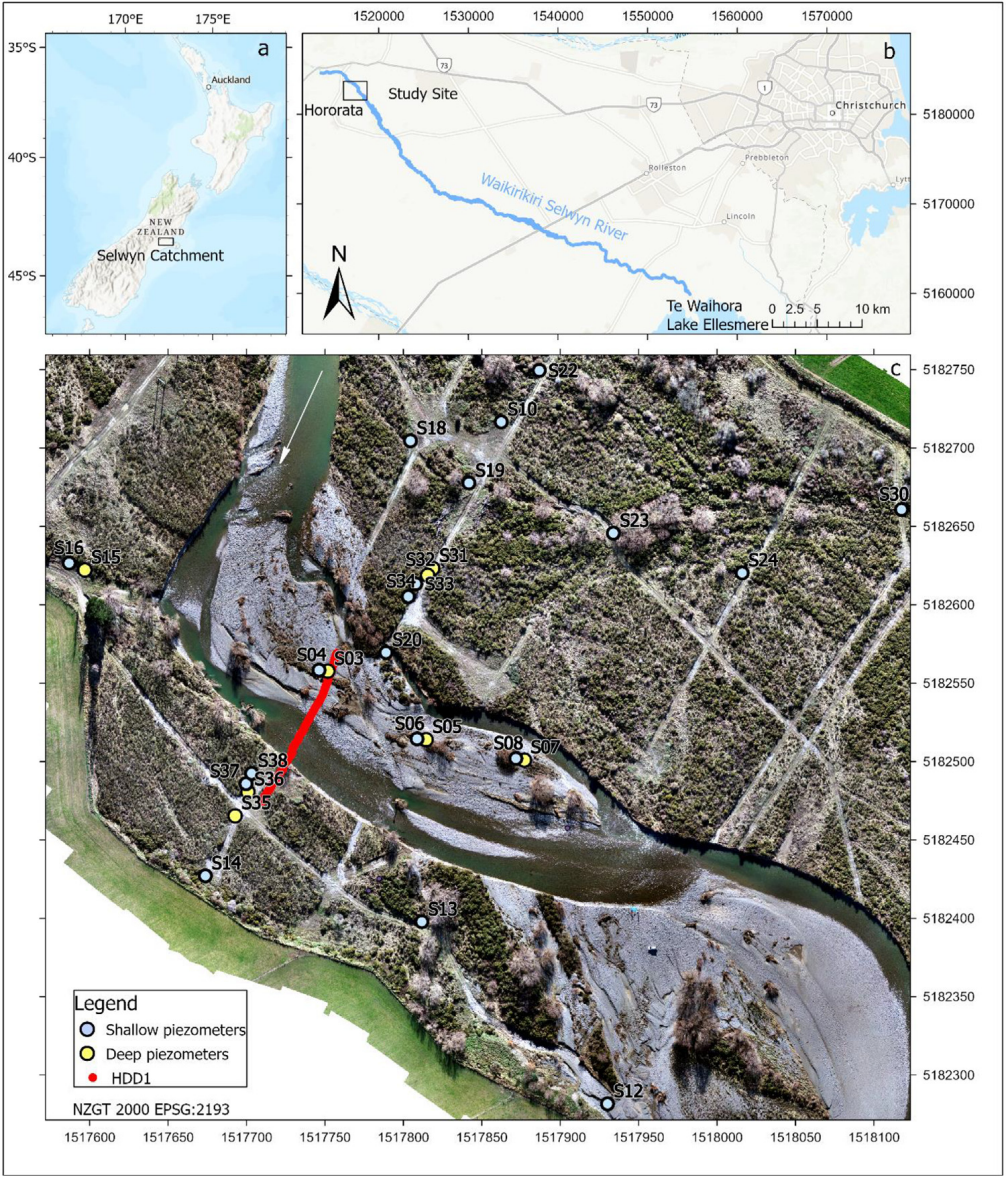
(a) Location of the Waikirikiri Selwyn River catchment, New Zealand, (b) Waikirikiri Selwyn River catchment and (c) Study site and the location of the buried A-DTS cable HDD1 and the network of shallow and deep piezometers.

Accompanying the shape file is a spreadsheet entitled ‘cable_location.xlsx’ which contains the x, y, z and z’ coordinates of the subsurface buried cable, HDD1, as well as the overlying ground surface. The first column, x, contains the northing coordinates, the second column, y, contains the easting coordinates, the third column, z, contains the elevation in meters above mean sea level (m amsl) of the cable, and the fourth column, z’, contains the elevation (m amsl) of the overlying ground surface directly above the cable (Fig. 1).

## 3 Experimental Design, Materials and Methods

**Fig. 2:**
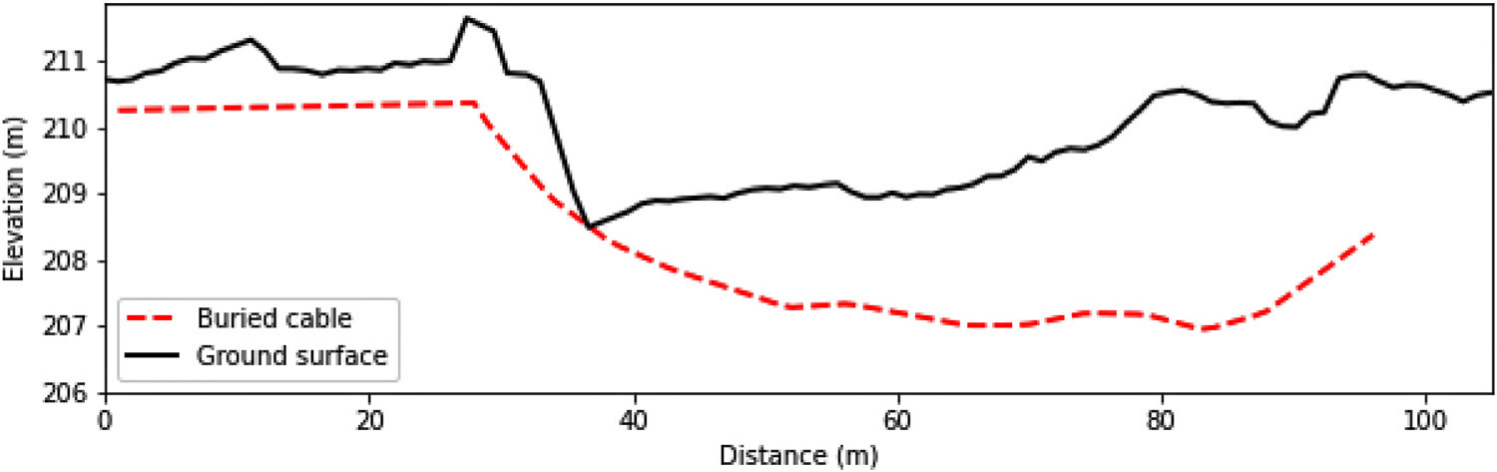
Cross-sectional profile showing the buried hybrid fiber optic cable, relative to the ground surface.

Horizontal directional drilling was used at the Waikirikiri Selwyn River study site to drill a 100 m long 125-mm drillhole to approximately 5 m depth from the true right bank of the river, running perpendicular to the river direction (Fig. 2). When the drilling was complete, the hole was filled with drilling mud and a temporary 125-mm diameter poly casing was attached to the drill bit, and back-reamed through the 100 m long drillhole. This was followed by pulling the hybrid fiber optic cable back through the casing into position and then the temporary casing was removed, causing the surrounding sediments to collapse around and against the cable. To protect the cable from damage by the river, the cable connector terminals were housed in a secure concrete pipe on the riverbank [1].

The hybrid FO cable contained both fiber optic cables for temperature sensing and copper conductors for heating. A Silixa XT-DTS sensor combined with a Silixa Heat Pulse System was used to conduct active-distributed temperature sensing (A-DTS) on the hybrid cable. The DTS unit has a sample interval of 0.25 m, with a spatial resolution of 0.54 m and a temperature resolution of 0.01 °C [4]. An acquisition sampling rate of 12 s was used.

For calibration purposes, two 8 m sections of the cable were placed in calibration baths, one containing ambient temperature river water (∼10 °C) and another containing ice water (0 °C). These baths were equipped with independent Pt-100 temperature probes to calibrate the DTS and ensure measurement accuracy.

The experiment was conducted when the river stage and flow height were relatively stable to assess temporal variation in groundwater recharge. The experiment was conducted over a 24-hour period and comprised twelve back-to-back surveys each measuring the ambient temperature for 300 s, followed by 3600 s heating the cable with 15 W m^−1^ power applied and 3300 s measuring the temperature decrease.

## Limitations

The main objective of this study was to validate the methodology and installation of A-DTS and how it can be used to calculate groundwater recharge beneath braided river systems. The dataset was not used to investigate seasonal variations but to conduct a series of A-DTS cycles over a 24-h period during stable and low flow river conditions and evaluate how these individual surveys compared to one another.

## Ethics Statement

This study did not affect any human or animal subjects.

## CRediT authorship contribution statement

**Alice J. Sai Louie:** Conceptualization, Methodology, Investigation, Data curation, Formal analysis, Visualization, Writing – original draft. **Leanne K. Morgan:** Conceptualization, Methodology, Writing – review & editing, Supervision. **Eddie W. Banks:** Conceptualization, Methodology, Investigation, Writing – review & editing, Supervision. **David Dempsey:** Software, Formal analysis, Writing – review & editing, Supervision. **Scott Wilson:** Conceptualization, Funding acquisition, Writing – review & editing, Supervision.

## Data Availability

Active-Distributed Temperature Sensing data collected beneath a braided river for quantification of groundwater recharge (Original data) (Mendeley Data)
